# Osteosarcoma of the lower limb metastasized to the septum and right side of the heart: a case report

**DOI:** 10.1186/s13256-017-1325-0

**Published:** 2017-06-13

**Authors:** Elisha Osati, Alice Kaijage, Reuben Muta, Peter Muhoka, Merckris Mango, Peter Kisenge, Bashir Nyangasa, Pedro Pallangyo, Delillah Kimambo, Mohamed Janabi

**Affiliations:** 1 The Jakaya Kikwete Cardiac Institute, P.O. Box 65141, Dar es Salaam, Tanzania; 2 Muhimbili Orthopaedic Institute, Dar es Salaam, Tanzania; 3 Muhimbili University of Health and Allied Sciences, Dar es Salaam, Tanzania

**Keywords:** Cardiac metastasis, Cardiac osteosarcoma, Echocardiography, CT, MRI

## Abstract

**Background:**

Metastatic cardiac tumors are far more common than primary tumors. Although the hematogenous spread of osteosarcoma is well known, the imaging findings of cardiovascular involvement by osteosarcoma are seldom reported and can be difficult to recognize.

**Case presentation:**

A 21-year-old man of African descent presented to our center complaining of shortness of breath, awareness of heart beats, easy fatigability, swelling of lower limbs, and left-side chest discomfort for the past 6 months getting worse for the last 3 months prior to his third readmission. In 2004 he was admitted with the diagnosis of osteosarcoma of his left calcaneus bone confirmed by bone biopsy and treated at an oncology center with several cycles of radiotherapy and chemotherapy; he was declared cured after 5 years of annual clinical and radiological skeletal survey follow-ups. In the current admission, a physical examination revealed bilateral lower limb swelling, pansystolic murmur on the left side of his sternum at fourth intercostal space (tricuspid area) grade three, hepatomegaly with a liver span of 17 cm, and a positive fluid test and shifting dullness.

**Conclusions:**

This case report presents a 21-year-old man with relapsed osteosarcoma manifesting as metastatic lesions to his right ventricle encroaching on his interventricular septum, which was identified by transthoracic/transesophageal echocardiography computed tomography scan and cardiac magnetic resonance imaging.

## Background

Although hematogenous spread of osteosarcoma is well known, the imaging findings of cardiovascular involvement by osteosarcoma are seldom reported and can be difficult to recognize. The enhanced resolution of modern computed tomography (CT) and magnetic resonance imaging (MRI) scanners may lead to better detection of cardiovascular involvement [1].

The antemortem diagnosis of cardiac metastases in osteogenic sarcoma has been rarely documented whereas a prevalence as high as 20% is seen at autopsy, suggesting that cardiac involvement is a late stage complication. As the survival times for primary osteosarcoma continue to improve, late stage complications will undoubtedly become more prevalent in the future [2, 3].

## Case presentation

**Fig. 1:**
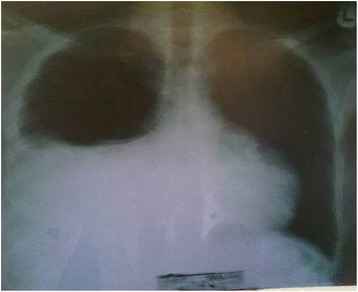
Chest X-ray showing right-sided pleural effusion and cardiomegaly

**Fig. 2:**
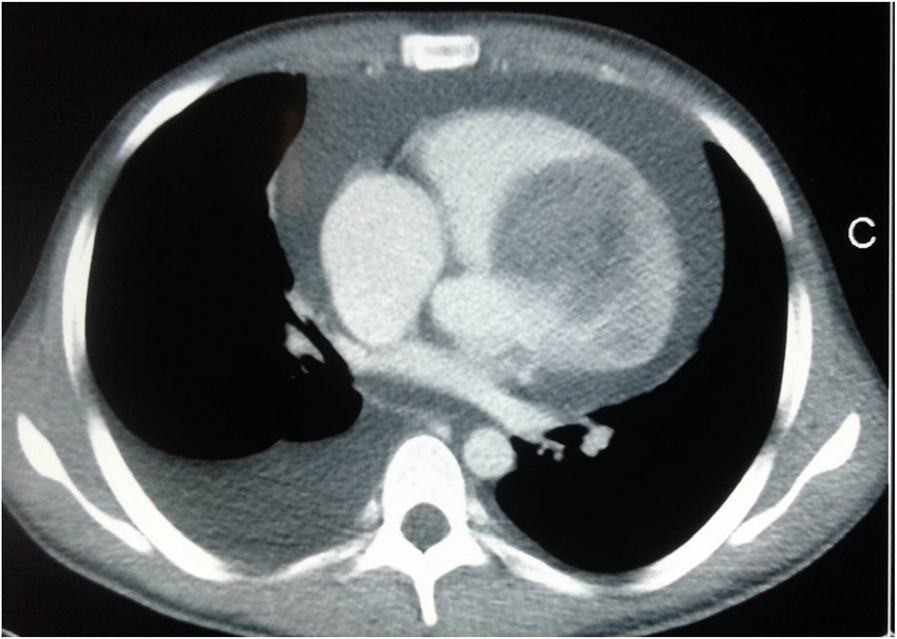
Chest computed tomography scan showing right ventricular mass, pericardial effusion, and pleural effusion

**Fig. 3:**
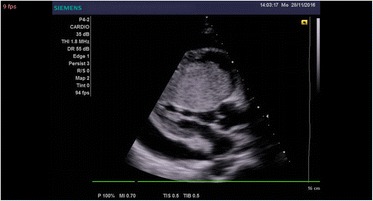
Transthoracic echocardiography showing right ventricular mass and pericardial effusion

**Fig. 4:**
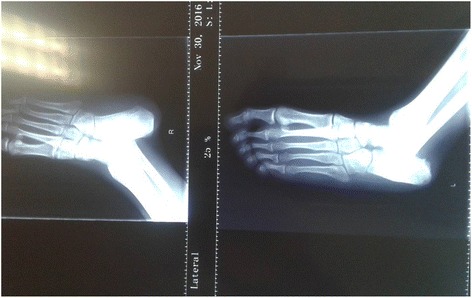
X-ray of the left calcaneus bone showing the bone erosion following osteosarcoma

A 21-year-old man of African descent presented to our center complaining of shortness of breath, awareness of heart beats, easy fatigability, swelling of lower limbs, and left-side chest discomfort for the past 6 months getting worse the last 3 months prior to this third readmission. Such symptoms started gradually from 2015, approximately 6 years after he had received a declaration that his osteosarcoma had been cured. In 2004 he was admitted with the diagnosis of osteosarcoma of his left calcaneus bone, confirmed by bone biopsy, and treated at an oncology center with several cycles of radiotherapy and chemotherapy; he was declared cured after 5 years of annual clinical and radiological skeletal survey follow-ups. In the current admission, a physical examination revealed bilateral lower limb swelling, pansystolic murmur on the left side of his sternum at fourth intercostal space (tricuspid area) grade three, hepatomegaly with a liver span of 17 cm, and a positive fluid test and shifting dullness. Biochemical investigations noted elevated levels of brain natriuretic peptide. An electrocardiogram demonstrated an abnormal analysis. A chest X-ray showed cardiomegaly with increased interstitial pulmonary markings and pleural effusion (Fig. 1). A CT scan of his chest revealed cardiomegaly with right ventricular and right atrial emphasis; a non-enhanced mass measuring 5.99×5.59 cm was noted in his right ventricle (RV). His aorta (ascending, arch, and thoracic) was preserved. Marked pericardial effusion was noted with no septations. There was marked right pleural effusion; thoracic cage and soft tissue were maintained. The visualized portion of his abdomen revealed liver congestion ascites and suspicious filling defects in his inferior vena cava (IVC; Fig. 2). Echocardiography revealed a medium echogenic mass in his RV with rich blood flow signal; it had a maximum thickness of 4.32×6.60 cm, occupying right ventricular outflow tract and encroaching on the interventricular septum (Fig. 3). However, systolic function was preserved (ejection fraction 65%). An X-ray of his left calcaneus bone showed an erosion process (Fig. 4). Pleural fluid cytology revealed benign epithelia cells in small clusters.

Therapy was focused on improving the New York Heart Association (NYHA) class III heart failure which he presented on admission. He was discharged on 15 December 2016 in a fair condition with NYHA class II and booked in a follow-up clinic by the time of drafting this manuscript.

## Discussion

Noncardiac tumors may invade the heart by means of lymphatic or hematogenous dissemination, local extension, or a transvenous route. However, catheter-based biopsy is not suitable for cardiac tumors. Therefore, it is extremely important to accurately determine the nature of the cardiac mass before considering surgical options [4]. Our patient presented with features suggestive of heart failure: clinical symptoms, signs, and laboratory examinations. A battery of imaging modalities used in this study revealed a mass in his RV extending to the interventricular septum. Hence, we illustrate the distinguishing characteristics and clinical presentation, as well as draw conclusions regarding the investigation and management of such rare cases. The demographic differences shown in this case include young male sex at the time of diagnosis and longer interval to the onset of the cardiac involvement. The clinical characteristics involve hemodynamic compromise or precordial abnormality, with MRI emerging as the gold standard for diagnosis. Cardiac involvement is a strong predictor of disease elsewhere and mandates careful surveillance; our patient had osteosarcoma diagnosed 12 years ago, at the age of nine [3]. Differential diagnosis included tumor or thrombus. Transesophageal echocardiography confirmed that the mass was attached to the RV free wall with a broad base. Cardiac MRI with and without contrast was done, it showed a large mass in his RV. The MRI features were consistent with tumorous involvement of the RV. Unfamiliarity with the imaging features may result in under-recognition and misinterpretation of intravascular tumor thrombus as bland thrombus. This emphasizes the need for a multidisciplinary approach as applied in this case. Anticoagulation must be initiated in such patients with proven thrombus on top of the tumor by biopsy. There is also debate about the best time to intervene surgically in this clinical scenario. The medical panel (including cardiologists, cardiothoracic surgeons, radiologist, and oncologists) together with the family agreed that the risks of attempting a cardiac biopsy in the patient's current state outweighs the potential benefits. Our patient was discharged to continue a conservative approach.

A limitation is that our center does not perform ^18^F-fluorodeoxyglucose (^18^F-FDG) positron emission tomography (PET)/CT which could have revealed the morphological features of the cardiac tumor and glucose uptake; these could have been significant indicators of tumor malignancy and the whole body metastasizes [5]. Metastases to the heart occur late during malignancy. The heart is rarely the first site of malignant disease.

## Conclusions

This report presents a 21-year-old man with relapsed osteosarcoma manifesting as metastatic lesions to his RV encroaching on his interventricular septum, which was identified by transthoracic/transesophageal echocardiography CT scan and cardiac MRI.
